# Permafrost Active Layer Microbes From Ny Ålesund, Svalbard (79°N) Show Autotrophic and Heterotrophic Metabolisms With Diverse Carbon-Degrading Enzymes

**DOI:** 10.3389/fmicb.2021.757812

**Published:** 2022-02-03

**Authors:** Katie Sipes, Raegan Paul, Aubrey Fine, Peibo Li, Renxing Liang, Julia Boike, Tullis C. Onstott, Tatiana A. Vishnivetskaya, Sean Schaeffer, Karen G. Lloyd

**Affiliations:** ^1^Microbiology Department, University of Tennessee, Knoxville, Knoxville, TN, United States; ^2^Department of Biosystems Engineering and Soil Science, University of Tennessee, Knoxville, Knoxville, TN, United States; ^3^Department of Geosciences, Princeton University, Princeton, NJ, United States; ^4^Alfred Wegener Institute, Potsdam, Germany; ^5^Geography Department, Humboldt-Universität zu Berlin, Berlin, Germany; ^6^Center for Environmental Biotechnology, University of Tennessee, Knoxville, Knoxville, TN, United States

**Keywords:** Svalbard, permafrost, active layer, carbon, nitrogen, metagenome, cultures, enzymes

## Abstract

The active layer of permafrost in Ny Ålesund, Svalbard (79°N) around the Bayelva River in the Leirhaugen glacier moraine is measured as a small net carbon sink at the brink of becoming a carbon source. In many permafrost-dominating ecosystems, microbes in the active layers have been shown to drive organic matter degradation and greenhouse gas production, creating positive feedback on climate change. However, the microbial metabolisms linking the environmental geochemical processes and the populations that perform them have not been fully characterized. In this paper, we present geochemical, enzymatic, and isotopic data paired with 10 *Pseudomonas* sp. cultures and metagenomic libraries of two active layer soil cores (BPF1 and BPF2) from Ny Ålesund, Svalbard, (79°N). Relative to BPF1, BPF2 had statistically higher C/N ratios (15 ± 1 for BPF1 vs. 29 ± 10 for BPF2; *n* = 30, *p* < 10^–5^), statistically lower organic carbon (2% ± 0.6% for BPF1 vs. 1.6% ± 0.4% for BPF2, *p* < 0.02), statistically lower nitrogen (0.1% ± 0.03% for BPF1 vs. 0.07% ± 0.02% for BPF2, *p* < 10^–6^). The d^13^C values for inorganic carbon did not correlate with those of organic carbon in BPF2, suggesting lower heterotrophic respiration. An increase in the δ^13^C of inorganic carbon with depth either reflects an autotrophic signal or mixing between a heterotrophic source at the surface and a lithotrophic source at depth. Potential enzyme activity of xylosidase and N-acetyl-β-D-glucosaminidase increases twofold at 15°C, relative to 25°C, indicating cold adaptation in the cultures and bulk soil. Potential enzyme activity of leucine aminopeptidase across soils and cultures was two orders of magnitude higher than other tested enzymes, implying that organisms use leucine as a nitrogen and carbon source in this nutrient-limited environment. Besides demonstrating large variability in carbon compositions of permafrost active layer soils only ∼84 m apart, results suggest that the Svalbard active layer microbes are often limited by organic carbon or nitrogen availability and have adaptations to the current environment, and metabolic flexibility to adapt to the warming climate.

## Introduction

Temperatures in the Arctic are increasing faster than they are at lower latitudes (Cohen et al., 2014; Pörtner et al., 2019). The permafrost of Svalbard, in particular, is known as “warm permafrost,” since it is close to 0°C, making it very sensitive to warming (Keating et al., 2018). Globally, permafrost and active layer soils are estimated to contain carbon stocks that are twice as large as the current atmospheric carbon pool (Mann et al., 2014). Models have estimated about 1,600 Pg (10^15^) of carbon in permafrost regions (Waldrop et al., 2010), with an estimated 195 Pg of carbon projected to be released in the form of gaseous carbon compounds by the year 2100, which would increase global temperatures an additional 0.03–0.23°C (Beermann et al., 2017; Salmon et al., 2018). Microbial activity within soils, especially in the active layer that thaws every summer, is an important driver of nutrient and carbon cycling (Mackelprang et al., 2011). Future climatic conditions are likely to increase the availability of carbon sources for microbial decomposition since temperature, water availability, and the rate of microbial carbon degradation in permafrost are related (Waldrop et al., 2010; Beermann et al., 2017; Garnello et al., 2021). The permafrost of the high Svalbard Arctic (79°N) is currently a small net annual sink for CO_2_ (Jentzsch et al., 2021). Nitrogen is often a limiting reagent for plant growth and microbial metabolism in the Arctic and is derived from the decomposition of active layer organic matter (Schimel and Schaeffer, 2012; Salmon et al., 2018). Pathways for carbon degradation and the relationship of carbon degradation to nitrogen limitation have not been fully characterized in this region nor have they been coupled to extracellular enzyme assays, isolations, and isotopic compositions of carbon and nitrogen. Due to increases in temperature and microbial activity, this warm permafrost location could be at the brink of becoming a net source of greenhouse gasses such as CH_4_ and CO_2_. Here, we combined soil carbon and nitrogen analysis, extracellular enzymes assays, isolate activities, and metagenomes to gain a broader view of current carbon degradation activities, and their relationship to nitrogen cycling, in Svalbard active layer soils.

The permafrost at the site near the Bayelva River in Ny Ålesund, Svalbard (79°N), has been continuously monitored for physical characteristics of the soil and snow since 1998 and reports a yearly average permafrost temperature of –2.5°C (Boike et al., 2018). Microbial interactions with the geochemical processes of the active layer have not yet been characterized at this site. However, analysis of nearby snow cover and fjord sediment found *Alphaproteobacteria*, *Betaproteobacteria*, *Gammaproteobacteria*, *Firmicutes*, and *Actinobacteria* to be the most common phyla (Amato et al., 2007; Buongiorno et al., 2019, 2020). Annual variation in active layer soil microbial communities in Adventdalen, Svalbard (78°N), a site that is ∼615 km away from our study location, was observed to be largely driven by organic matter availability or sunlight (Schostag et al., 2015). Metagenomic and gas flux analyses at Adventdalen showed that the active-layer microbial community is involved in many different carbohydrate degradation pathways (Müller et al., 2012; Xue et al., 2020). While total DNA population studies allow for a broad characterization of the microbial community, culturing environmental organisms can provide direct evidence of the interactions between organisms and the environment. Arctic conditions are difficult to replicate in a laboratory setting due to low nutrient availability and seasonal freezing. Commonly, cultures have been grown in a wide range of temperatures (+4 to +30°C) to parallel Arctic seasonal variations and to investigate ubiquitous organisms (Vishnivetskaya et al., 2000; Sonjak et al., 2006; Finster et al., 2009). One commonly used medium for cultivating Arctic soil isolates is R2A (Vishnivetskaya et al., 2000; Hansen et al., 2007; Finster et al., 2009; Beyer et al., 2015), which mimics the low-energy conditions of the active layer and has been found to be best suited for the isolation of slow growing oligotrophic organisms (Vishnivetskaya et al., 2000; Medina et al., 2017).

We investigated the microbial influence on two active layer geochemical profiles by combining cultured isolates with metagenomic inferences, extracellular enzyme assays, and soil geochemistry such as carbon and nitrogen content and stable isotope ratios. This multifaceted approach showed heterotrophic metabolism dominating the location with higher labile carbon. Autotrophic signatures are more prevalent in the site with higher inorganic carbon content and a higher C/N ratio. Potential rates of extracellular enzymes and the gene counts of the enzymes were compared between measurements made on bulk soil vs. 10 *Pseudomonas* sp. isolates. Enzymatic analyses over a range of temperatures displayed higher activity in colder temperatures in both the bulk soil and cultured isolates. Understanding the pathways of carbon degradation in natural microbial communities and cultured isolates from the active layer soils is important for determining how these communities will degrade natural organic matter, as more of it becomes available due to thaw.

## Materials and Methods

### Field Sampling

**FIGURE 1:**
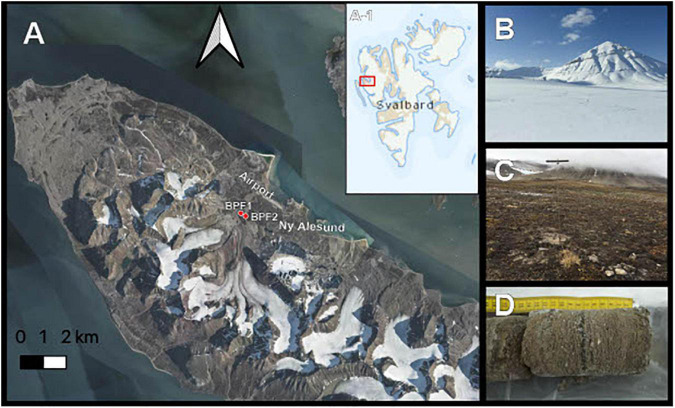
Sample site. **(A)** Active layer cores were taken from two permafrost sites near the Bayelva River in the Leirhaugen glacier moraine in Ny Ålesund, Svalbard, 79°N. Inset A-1 shows the location of the image in panel **(A)** within the Svalbard archipelago. **(B)** Picture taken from the BPF1 borehole in April 2018 at the time of retrieval. **(C)** Borehole location of BPF1 marked by metal permafrost probe in September 2019. **(D)** Example of core sample retrieved from BPF2 site.

In April 2018, soil core samples were drilled from completely frozen active-layer permafrost in Ny Ålesund, Svalbard (Figure 1). These two drill sites were near Bayelva Monitoring Site (Boike et al., 2018). Bayelva Permafrost Site 1 (BPF1) (N 78° 55.237′ E 011° 50.495′) is closest to the Bayelva Monitoring Site, 21 m above sea level. BPF2 (∼84 m from BPF1, N 78° 55.261′ E 011° 50.294′) is near a summer glacial melt riverbank at an elevation of 20 m above sea level (Figure 1A). Three boreholes were drilled at each of the two sites with a SIPRE auger drill (NSF, United States) with ∼0.6 m snow cover that was shoveled away prior to drilling (Figures 1B,C). Depths of core samples were limited by the ability to recover intact material. Core depths for BPF1 were 21, 20, and 58 cm, and core depths from BPF2 were 30, 21, and 21 cm below the surface (Figure 1D). Freshly cored samples were removed from the drill and kept inside presterilized polycarbonate core liners (Jon’s Machine Shop, United States) used during drilling. Core liners were capped and stored inside a sterile lined cooler to maintain frozen temperature.

### Core Processing

Cores were removed from the core liner and sliced into 2-cm-depth intervals using a sterile geological sampling hammer and chisel at the King’s Bay AS Marine Laboratory (Ny Ålesund, Svalbard). A portion of the sample was weighed and then dried in a 60°C oven for 24 h to determine gravimetric water content. Bulk density of the cores was estimated by measuring volume and dry mass of one intact 2.8-cm core puck with a diameter of 8 cm collected from BPF1; the resulting uniform bulk density was applied to all core samples for bulk density.

### Culturing

To study colony variation and CFU/ml, we tested various soil dilutions and three different agar types on the BPF1 0–2 cm. Following previously published methods (Vishnivetskaya et al., 2000), we tested R2A, TSA, and 1/2-strength TSA with a Master Soil Mixture (MSM) made from 10 g soil and 100 ml of 1 × phosphate-buffered saline (PBS, pH 7.2). The following four final soil suspensions ratios were plated: MSM:1XPBS; 0.1:0.9 (1 ml plated), 0.5:0.5 (1 ml plated), 1:0 (1 ml plated), and 3:0 (3 ml plated) for each of the three media types, in triplicate. CFUs/ml were counted after growth at 4°C for 3 weeks. Full-strength TSA yielded a lawn of colonies after just a few days, and 1/2 TSA yielded only a few colonies (Supplementary Figure 1). R2A agar was chosen for our study, since it had the greatest colony diversity and allowed for slower growing colonies to form. The MSM:PBS dilutions listed above were made for eight depth intervals between BPF1 and BPF2 core sites (Supplementary Figure 2). From BPF1, the depth intervals were 0–12 cm, 12–24 cm, 24–36 cm, 36–48 cm, and 48–58 cm. From BPF2, the depth intervals were 0–12 cm, 12–20 cm, and 20–30 cm. An MSM from these eight depth intervals were plated with the same soil suspension scheme and incubated at 4°C for 3 weeks. After CFU/ml and colony variants were measured on the experimental plates, we streaked for isolation on R2A. Of the hundreds of isolates obtained on plates, 10 were selected for continued analysis based on their morphological diversity. Isolates were grown on R2A for 4 weeks at 4°C, after choosing R2A over TSA and 1/2-strength TSA because the diluted medium allowed for isolation of oligotrophic microbes (Supplementary Figures 1, 2). The exact depth intervals they came from are listed in Table 1.

**TABLE 1:** Closest relative of 16S rRNA genes from cultured *Pseudomonas* spp. isolated from bulk soil.

Sample	Svalbard site origin	Percent match	Organism name	Location	First author	NCBI reference
*B3*	BPF1 24–36 cm	99%	*Pseudomonas silesiensis* strain ILQ215	Various soil samples from The Peruvian Andean Plateau	Carolina Chumpitaz-Segovia	(Published) June 23rd, 2020
*E5*	BPF1 36–48 cm	100%	*Pseudomonas* sp. strain PAMC 27331	Antarctic soil	H.J. Park	(Submitted) June 3rd, 2020
*G17*	BPF1 48–58 cm	99%	*Pseudomonas* sp. strain PAMC 27357	Frozen soil samples from Council, Alaska United States.	Hyoungseok Lee	(Sample collected) June 29, 2012
*B4*	BPF1 48–58 cm	99%	*Pseudomonas mandelii* strain JZY4-67	QinLing Mountain China	R. Chen	(Accepted) February 26th, 2020
*E6*	BPF1 0–12 cm	99%	*Pseudomonas mandelii* strain UTB_118	Sediment samples from Station Juan Carlos I., Livingston Island, Antarctica	L. Ward-Bowie	(Sample collected) February 25th, 2018
*G19*	BPF1 0–12 cm	99%	*Pseudomonas* sp. strain E1-4	Antarctic soil	M. Zhou	(Submitted) January 18th, 2017
*B5*	BPF1 0–12 cm	100%	*Pseudomonas* sp. strain PAMC 27292	Antarctic soil	H.J. Park	(Submitted) June 3rd, 2020
*E7*	BPF2 0–12 cm	100%	*Pseudomonas* sp. strain PAMC 27303	Antarctic soil	H.J. Park	(Submitted) June 3rd, 2020
*B7*	BPF2 20–30 cm	100%	*Pseudomonas mandelii strain BLH-Y1*	Qinghai-Tibet Plateau	Y. Wang	(Submitted) September 24th, 2013
*G16*	BPF1 48–58 cm	99%	*Pseudomonas mandelii* strain UTB_115	Sediment samples from Station Juan Carlos I., Livingston Island, Antarctica	L. Ward-Bowie	(Sample collected) February 25th, 2018

### Soil Geochemical Analyses

Total carbon, nitrogen, carbon isotopic signature (δ^13^C), and nitrogen isotopic signature (δ^15^N) were determined on completely dried soil that was ground using a mortar and pestle into a fine powder. Large stones were removed, and the final particle size of the fine powder was not measured before analysis. To quantify the organic fraction of carbon, 1 ml of 1 N HCl was added to 5.0 g of soil and oven dried at 45°C for 56 h to volatilize inorganic carbon (Harris et al., 2001). The δ^13^C and percentage of inorganic carbon were calculated through mass balance for samples with > 0.7% inorganic carbon (Komada et al., 2008). Total carbon, nitrogen, δ^13^C, and δ^15^N were determined on finely ground soil samples (30 mg for BPF1 and 40 mg for BPF2) using a Costech ECS4010 Elemental Analyzer coupled to a Thermo-Finnigan Delta+XL mass spectrometer via a Thermo-Finnigan Conflo III device. Helium was used as the carrier gas, and the oxidation furnace was operated at 1,050°C and the reduction furnace at 650°C. These measurements were performed at the Stable Isotope Laboratory at the University of Tennessee, Knoxville, United States.

Extractable dissolved organic carbon (DOC), phosphate, and inorganic nitrogen were measured on each of the 2-cm intervals of core samples (Supplementary Figure 3). Extracts were prepared by combining 5.0 g soil with 20 ml of 0.5 M potassium sulfate and shaking at room temperature for 4 h. Samples were then filtered through a Whatman GF/B glass microfiber filter (1.0 μm pore size) using a vacuum extraction manifold. Filtrate was collected and frozen at –20°C for 12 h. DOC was quantified by reacting extracts with a 0.42 M potassium persulfate (Doyle et al., 2004) to oxidize soil organic carbon to CO_2_. A series of potassium hydrogen phthalate standards were included in each analysis to use for calculation of persulfate-oxidized organic carbon. Samples and standards were reacted overnight (80°C) in sealed glass vials with rubber septa for headspace gas sampling, which was conducted after samples had cooled to room temperature using an infrared CO_2_ gas analyzer. Sample extracts were carried through three colorimetric assays to measure extractable phosphate (PO_4_^3–^), nitrate (NO_3_^–^), and ammonium (NH_4_^+^) concentrations. The sum of nitrate and ammonium concentrations is used to calculate total inorganic nitrogen, and the sum of organic nitrogen and inorganic nitrogen is equal to total nitrogen. Inorganic phosphate was measured using the Malachite Green assay (D’Angelo et al., 2001). Nitrate was determined using a vanadium (III) chloride reagent (Doane and Horwáth, 2003), and ammonium was quantified using the Berthelot reaction (Rhine et al., 1998). All nitrogen and phosphorous assays were conducted using protocols modified for a 96-well microplate reader (Synergy H1 Hybrid Reader, Biotek Inc., Winooski, VT, United States).

Additional measurements for electrical conductivity, pH, and labile carbon were performed on a separate core sample from each site that was divided as follows: BPF1, 0–12 cm, 12–24 cm, 24–36 cm, 36–48 cm, and 48–58 cm; BPF2, 0–12 cm, 12–20 cm, and 20–30 cm (Supplementary Figure 3). Electrical conductivity and pH were measured on these samples using calibrated bench-top meters (15 g soil in 45 ml deionized water). Additionally, pH, labile carbon, and electrical conductivity measurements were performed on eight soil sample increments (BPF1, 0–12 cm, 12–24 cm, 24–36 cm, 36–48 cm, 48–58 cm; BPF2, 0–12 cm, 12–20 cm, and 20–30 cm) using calibrated bench-top meters (15 g dry soil in 45 ml deionized water). Permanganate oxidizable carbon (POXC), used to analyze total labile carbon, was extracted and quantified according to the method of Weil et al. (2003). Briefly, 2.5 g was reacted with 20 ml 0.02 M potassium permanganate (KMnO_4_) + 0.1 M calcium chloride solution by shaking at 120 rpm for 2 min. After settling, extracts were diluted 1:100 with milliQ water then measured for absorbance at 550 nm wavelength using a microplate spectrophotometer. Standards of a known concentration of KMnO_4_ were included with each plate and used to determine the moles of KMnO_4_ oxidized upon reaction with soil organic carbon. Assuming 9,000 mg carbon oxidized per mole KMnO_4_ (Weil et al., 2003), the amount of POXC was corrected for soil water content and reported in μg POXC g dry soil^–1^.

### DNA Extraction and 16S rRNA Amplification

For DNA extraction from isolates, a pellet was formed with low-speed centrifuging (7,000 × *g*) of isolates that had been grown on R2A at 4°C for 4 weeks, and DNA was extracted with a Qiagen DNA Power Soil Kit (Qiagen, Germany). To amplify the 16S rRNA gene of the isolates, a PCR Master Mix was prepared as follows: 0.25 μl of Speedstar Taq polymerase (TaKaRa Bio, United States), 4 μl of 2.5 mM deoxyribonucleotide triphosphates (dNTPs), 5 μl of 10 × Fast Buffer 1 (TaKaRa Bio, United States), 10 μl of 27F primer (5′-AGAGTTTGATYMTGGCTCAG-3′) 10 μl of 1492R primers (5′-TACGGYTACCTTGTTACACTT-3′) (Frank et al., 2008) (Eurofins Genomics, United States), 29.25 μl of dH_2_O, and 2 μl of DNA, totaling 200 μl of volume per sample. Samples underwent PCR thermocycling in a BioRad T100 ThermoCycler (BioRad, United States) for 95°C for 1 min, 95°C for 5 s, and 65°C for 20 s. The last two steps were repeated 34 times. After the thermocycler, samples were dyed with 6 × TriTrack DNA Loading Dye (Thermo Fisher Scientific, United States). Visualization of the PCR product was compared to a GeneRuler 1 kb Plus DNA Ladder (Thermo Fisher Scientific, United States). The samples were then placed into a 1.5% agarose gel with Midori green DNA stain at 90 V for 45 min with BioRad PowerPac Basic (BioRad, United States) for PCR product verification.

### Sanger Sequencing of 16S rRNA Genes of Isolates

Amplified 16S rRNA gene (27F, 1492R) PCR product was cleaned with Qiagen PCR Clean Up Kit (Qiagen, Germany) and Sanger sequenced at the Sequencing Core Facility at the University of Tennessee, Knoxville. Sequences were viewed, and forward and reverse reads were combined from the chromatogram in 4Peaks (v.1.7.2), and DECIPHER v2.17.1 (Wright et al., 2012) was used to check for chimeras. The combined sequences were analyzed in nucleotide BLAST (v2.11.0), and the closest related organisms were downloaded for comparison. Output sequences were classified with SILVA Sina (v1.2.11). SINA Alignment (v1.2.11) was also used to compare these isolates to 16S rRNA genes cataloged in the SSU database and to make a RAxML tree. All sequenced isolates have been deposited on National Center for Biotechnology Information (NCBI) GenBank accession numbers MZ773212–MZ773221.

### Whole Genome Sequencing

DNA extractions from the 10 cultured isolates were sequenced with an Illumina MiSeq V3, 600 cycles (2 × 300) at The University of Tennessee, Knoxville Center for Environmental Biotechnology. Whole genome sequences were retrieved from Illumina BaseSpace and were assembled with SPAdes v3.13.0 (Bankevich et al., 2012) on KBase (link in “Data Availability” section) (Arkin et al., 2018). Prokka v. 1.14.6 was used for annotations (Seemann, 2014). All whole genomes are available on NCBI accession number PRJNA649544.

### Metagenomes

DNA was extracted from the longest core sample from each site (BPF1, 58 cm; BPF2, 30 cm) using the Qiagen DNA PowerSoil DNeasy DNA Extraction kit (Qiagen, Germany) with 0.5 g of starting material at King’s Bay AS Marine Laboratory, no more than 3 h after removal from the ground. DNA was quantified on each 2 cm soil sample using a Qubit™ 4 Fluorometer (Thermo Fisher Scientific, United States). To get a total of 10 ng/μl for each metagenome, BPF1 samples were pooled into two groups (0–30 cm and 30–58 cm), and all BPF2 samples were pooled (0–30 cm). These three samples were sequenced on an Illumina MiSeq with a V3 flow cell, using a 600 Nextera cycle kit with 275–300 bases paired-end reads. Data were downloaded from Illumina’s BaseSpace platform and analyzed with KBase (Arkin et al., 2018) with default program settings. Forward and reverse fastq reads were assembled with 98% of the two reads surviving, and adapters were trimmed with Trimmomatic v0.36 (Bolger et al., 2014); then, all three metagenomes were assembled separately with MetaSPAdes v3.14.1 (Nurk et al., 2017) and annotated with Prokka v. 1.14.6 (Seemann, 2014). Metagenome libraries of soil samples and whole genomes of isolates were compared with read mapping in terms of “reads per kilobase per read library” through Bowtie2 (Langmead and Salzberg, 2012) and in-house python scripts.

### Cell Counts

The same protocol was used for cell counts of the bulk soil intervals and the cultures. The soil intervals used a dilution of 1:20 (1 × PBS: Soil suspension); the cultures were diluted based on visual opacity and spectrophotometer reading but did not exceed a 1:40 dilution (1 × PBS:culture broth) (see Supplementary Data File 1). 5 × SYBR gold stain was added and filtered on a vacuum Hoefer box on 0.2-μm Millipore round filters. Filters were adhered to microscope slides with Vecta Sheild©, and a cover slip was applied. A microscope used was a Zeiss Axio Imager M2 Epifluorescence Microscope (Oberkochen, Germany). Cells were counted in 30 random fields of view at 10 × magnification in the singular grid hemocytometer for eyepiece PL 10 × /23 in 23 mm × 23 mm. Total cell counts were calculated by the following equation:


(1) $$\frac{Cells}{mL}=\frac{\begin{array}{c} \bar{x_{cells}} \end{array}}{samplefiltered}*\frac{{\text{A}}_{\text{filter}}}{{\text{A}}_{\text{grid}}}*dilutionfactor$$

where $\begin{array}{c} \bar{x_{cells}} \end{array}$ is the average of the cells counted, sample filtered is the total sample used in the dilution, A_filter_ is the area of the Millipore 0.2 μm filter used, A_grid_ is the area of the hemocytometer grid, and dilution factor is the initial dilution factor of the sample:1 × PBS.

### Potential Enzyme Activity

#### Bulk Soil

Maximum potential activities for seven major carbon, nitrogen, and phosphorous hydrolytic enzymes were assayed at three incubation temperatures (5, 15, and 25°C) using fluorometric methods in triplicate (Saiya-Cork et al., 2002; Bell et al., 2013). Fluorescently labeled substrates were used to measure the activity of exogenously added small substrate proxies for the following enzymes: α-glucosidase (AG), β-glucosidase (BG), β-D-cellubiosidase (CB), leucine aminopeptidase (LAP), N-acetyl-β-D-glucosaminidase (NAG), phosphatase (PHOS), and β-xylosidase (XYL). The same eight soil increments (2.75 g) used to measure soil extractables (above) were suspended with 50 mM Tris buffer (pH 7.7) matching the measured mean sample pH, using high-speed blending for 60 s. Added fluorescent labels 7-amino-4-methylcoumarin (MUC) and 4-methylumbelliferone (MUB) were used for standardization. Soil slurries were incubated with MUC and MUB standards and labeled substrates (200 μl of 200 μM solution) for 3 h at 25°C, 6 h at 15°C, and 24 h at 5°C in triplicate. Fluorescence was measured using a microplate reader (Synergy H1 Hybrid Reader, Biotek Inc., Winooski, VT, United States) with 365 nm excitation wavelength and 450 nm emission wavelength set at optimal gain. Enzyme activity was calculated in nmol g dry soil^–1^ h^–1^, with higher activities indicating a greater amount of fluorescently labeled substrate that was degraded under the ideal conditions of incubation.

#### Pure Culture Enzyme Activity

We modified the above maximum enzymatic activity potential method to measure the 10 cultured isolates’ activity. Cultures grew in broth R2A for 24 h at 25°C prior to the experiment to reach exponential growth phase (Supplementary Figure 4). Cultures at this time were also used for a cell count to determine the number of cells that were in solution (Supplementary Figure 5A). Cultures were suspended in 35 ml 7.7 pH Tris buffer and then distributed across the 96-well plate and incubated with the small substrate proxies listed above.

## Results

### Soil Geochemical Analyses

**FIGURE 2:**
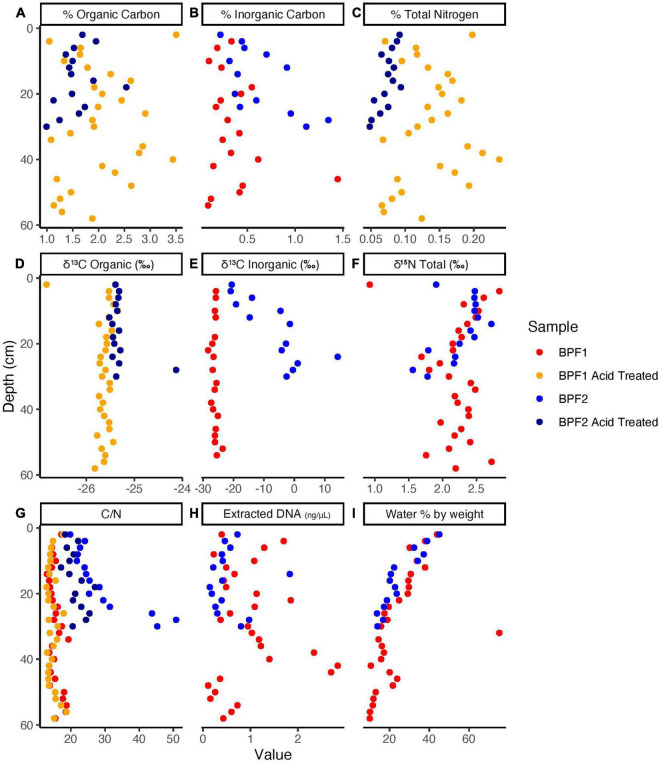
Elemental Analyzer data for BPF1 and BPF2 core samples before acid treatment (red for BPF1 and blue for BPF2) and after acid treatment (yellow for BPF1 and dark blue for BPF2).

**FIGURE 3:**
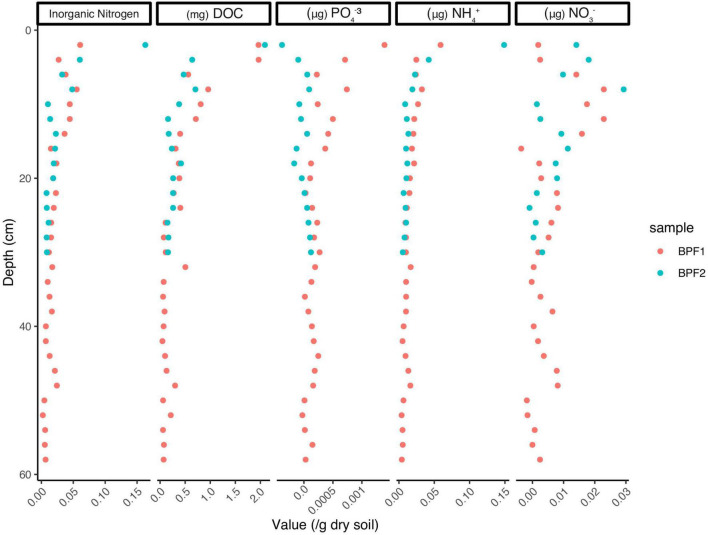
Values for PO_4_^3–^, NH_4_^+^, and NO_3_^–^ measured in μg/g of dry soil, with the exception of DOC measured in mg/g of dry soil. Inorganic nitrogen is the sum of NH_4_^+^ and NO_3_^–^. Values below zero indicate an amount below the detectable level.

The organic carbon of BPF1 ranged from 1 to 3.5% (Figure 2A), while inorganic carbon ranged between 0 and 0.5% (except for 1 point at 1.5%). BPF2 had a slightly decreasing trend with depth for organic carbon (2.0–1.0%) and slight increase in inorganic carbon from ∼0 to 1.5% with depth (Figure 2B and Supplementary Data File 1). Total nitrogen decreased with depth in BPF2 and ranged from 0.05 to 0.10%. Total nitrogen was nearly twice as high in BPF1, ranging from 0.05 to 0.25% (Figure 2C). The δ^13^C of organic carbon ranged from –26 to –25‰ with the exception of two points (Figure 2D). The inorganic δ^13^C values for BPF1 varied between –30% and –20‰, while the inorganic δ^13^C of BPF2 contains more ^13^C with depth (–20 to ∼10‰) (Figure 2E). δ^15^N in BPF1 and BPF2 decreased with depth (2.5–1.5‰) in the top 30 cm only (Figure 2F). Carbon to nitrogen ratios (C/N) in BPF1 ranged from ∼13 to 18, and those of BPF2 increased with depth from ∼19 to 50 (Figure 2G). The water content per weight of the samples decreased with depth at both sites, except for BPF1 30–32 cm, which was mostly ice (Figure 2I). Dissolved organic carbon (DOC) was similar in both cores, ranging from 0.3 to 3.2 mg/g_*dry soil*_. Inorganic nitrogen (sum of the measured NH_4_^+^ and NO_3_^–^) ranged from 0.05 to 0.2 μg/g_*dry soil*_ and was similar between the two cores (Figure 3). BPF2 had a lower concentration of PO_4_^3–^ at each depth compared to BPF1. However, both had negative values that were below the limit of detection of 0.0015 μg/g_*dry soil*_. NH_4_^+^ was highest at the surface (0.07 μg/g_*dry soil*_ for BPF1 and 0.18 μg/g_*dry soil*_ for BPF2) and consistently close to 0.05 μg/g_*dry soil*_ for both cores below the surface. NO_3_^–^ was similar for both cores, varying from 0 to 0.04 μg/g_*dry soil*_ (Figure 3). The measured electrical conductivity was < 0.05 mS in BPF1 and ranged from 0.025 to 0.17 mS in BPF2 (Supplementary Figure 3). The pH range of BPF1 and BPF2 were both between 7.4 and 8.1 (Supplementary Figure 3). Labile carbon (mg POXC/g_*dry soil*_) was lower in BPF2 (200–250), while BPF1 had greater POXC and higher variability with depth (320–825) (Supplementary Figure 3).

### Cultured Isolates

**FIGURE 4:**
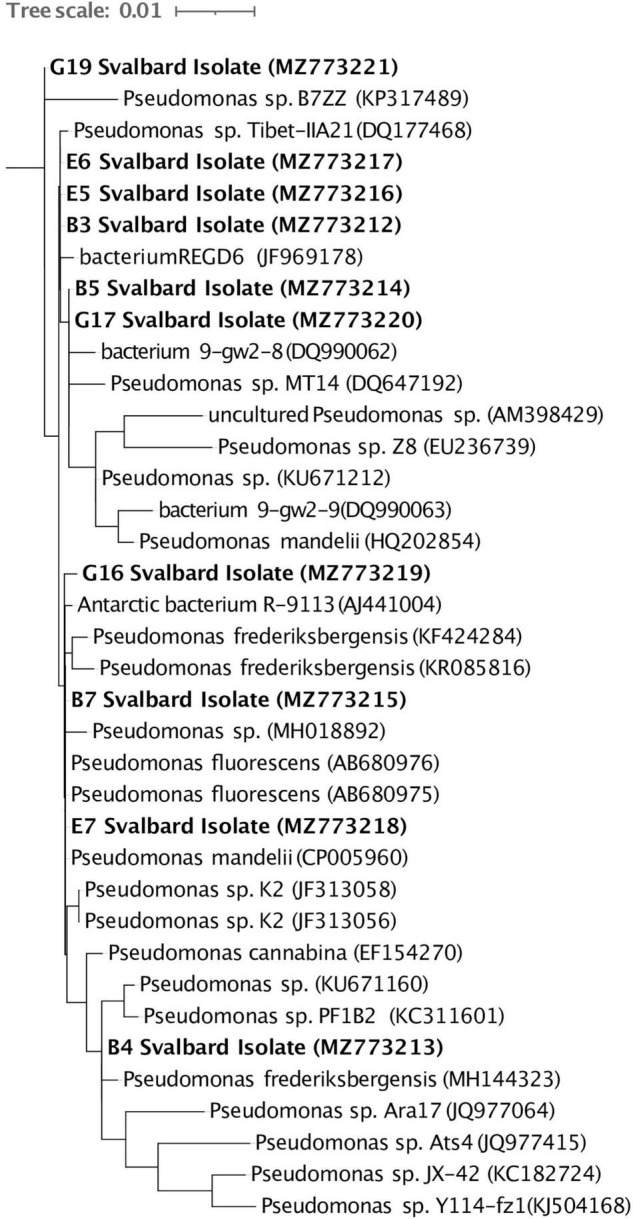
Full-length 16S rRNA gene sequences of the 10 Svalbard isolates (in bold) were aligned using the Silva SINA (v1.2.11), and Arb-Silva was used to identify their closest relatives. A RAxML tree was visualized in iTOL (v5.7). The GenBank accession number for each organism is listed in parentheses. The tree is rooted with *Aurantimonas* sp. (AB291857).

Ten cultures were chosen from the plated 0.5 ml soil suspension on R2A based on preliminary culture tests (see section “Materials and Methods,” Supplementary Figures 1, 2). Eight cultured isolates originated from BPF1 soil (Table 1) and two from BPF2 soil. All were Gram-negative rods of *Pseudomonas* sp. (Figure 4 and Supplementary Figure 6). Isolates of *Pseudomonas* sp. have been previously found in soil from Thuringian Basin, Council Alaska, Livingston Island Antarctica, and other Antarctica soils (Table 1 and references therein; Figure 4). The 16S rRNA gene sequence of each isolate was ≥ 99% similar to a previously cultured 16S rRNA genes on NCBI (Figure 4 and Table 1).

### Potential Enzyme Activities

#### Bulk Soil

**FIGURE 5:**
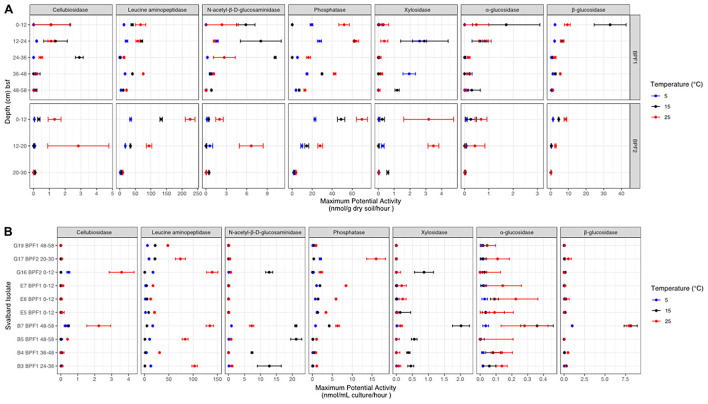
Potential enzyme activities at each temperature (red for 25°C, black for 15°C, and bright blue for 5°C) for **(A)** BPF1 and BPF2 and **(B)** 10 cultured isolates. Markers show the mean of triplicate measurements with error bars for one standard deviation. Enzymes are as follows: β-D-cellubiosidase (CB), leucine aminopeptidase (LAP), N-acetyl-β-D-glucosaminidase (NAG), phosphatase (PHOS), β-xylosidase (XYL), α-glucosidase (AG), and β-glucosidase (BG).

The highest maximum potential enzymatic activities in bulk soil were observed for LAP, PHOS, and BG, with the highest value from LAP at 25°C in BPF2 0–12 cm (225 nmol/g/h) (Figure 5 and Supplementary Table 1). The lowest maximum enzymatic potential activities were from AG, XYL, and CB. PHOS was the only enzyme of these three to have higher activities at 25°C for all eight soil intervals, with the other enzymes showing maximum potential activity at the lower 15°C temperature for at least one depth (Figure 5A). One sample (BPF1, 36–48 cm) had higher XYL activity at 5°C than either 15 or 25°C (∼2 nmol/g/h). In BPF1, NAG had the highest activity at 15°C for all intervals except 36–48 cm (∼1–10 nmol/g/h). In three depths of BPF1, XYL had a higher activity at the two lower temperatures. These increased activity at lower temperatures were only observed for BPF1. The 5°C treatment had the lowest enzyme activity for half of the tested soil intervals. BG in both sites had the lowest activity at 5°C. The 25°C treatment had the highest enzyme activity for 59% of the tested soil intervals. Temperature was correlated with LAP (spearman = 0.66) and PHOS (Spearman = 0.64) (Supplementary Figure 7). Most enzymatic activities decreased with depth at both sites, except for PHOS, LAP, and XYL in BPF1.

#### Culture Isolates

The 10 cultured *Pseudomonas* sp. isolates displayed a wide range of maximum potential enzymatic activities (Figure 5B and Supplementary Table 2). The highest activities were at 25°C from G16 (3.5 nmol/ml/h) and B7 (2.4 nmol/ml/h). NAG and XYL had higher activity at 15°C for five of the cultures. PHOS and LAP had higher activity for each cultured organism at 25°C. Culture E5 had low activities for all enzymatic substrates measured, most likely because it had much lower cell abundance than the other cultures (Supplementary Figure 5A). Compared to measurements in bulk soil, the cultures generally had higher maximum potential activities for NAG and lower values for PHOS, XYL, AG, and BG. The highest activities for cultures were in the LAP, NAG, and PHOS, in descending order. Overall, the 25°C treatment had the highest activity in most of the enzymes, except for XYL and NAG, similarly to the soil suspensions. CB, AG, and BG had very low activities in all cultures except B7, possibly because B7 had more cells than the other cultures at the time of measurement (Supplementary Figure 5A).

#### Metagenomes

Extracted DNA was very low throughout the 44 individual depth samples with only three samples higher than 2 ng/μl in BPF1 (Figure 2H). The three metagenomic libraries had between 8 million and 25 million reads that assembled into 7,000–13,000 contigs (Table 2). Since all metagenomic bins were within the MIMAGs low quality or medium quality standards (Bowers et al., 2017), we analyzed the whole metagenomic assembly as an indicator of the environmental abilities. Whole genome sequencing (WGS) was used to obtain genomes for the cultured isolates. Metagenomic reads were then mapped to the cultured whole genome isolates (Supplementary Figure 8). Genomes of the cultured isolates recruited reads from at least one metagenome (7–22,500 reads per kilobase per metagenomic library). G17 and G19 recruited the most reads from all three metagenomes. The 10 culture whole genomes and the three metagenome libraries were analyzed for presence of the genes encoding the enzymes tested in the enzymatic activity measurements (Table 3 and Supplementary Table 3). LAP had the highest range of gene counts (2–71), and PHOS had the most even distribution of counts between all the samples (9–,15). AG, BG, and NAG had little to no gene counts, while XYL had a sparse amount. Leucine aminopeptidase was the only enzyme that appeared to be related to the quantity of genes in the metagenomes. LAP had the cumulative highest number of gene counts and the highest range of activity for the bulk soil and the cultures at 25°C. PHOS genes were present in every sample and had highest activities in the 25°C treatment.

**TABLE 2:** MiSeq metagenome information.

BPF Metagenome library	BPF1 0–30 cm	BPF1 30–58 cm	BPF2 0–30 cm
*Number of reads*	25,871,532	8,366,690	13,769,728
*Number of contigs*	13,898	3,316	7,525
*Number of contigs > 10,000 bp*	612	299	186
*Number of contigs ≥ 100,000 bp*	0	0	0
*Largest contig*	82,493	66,148	35,326
*Total length*	52,430,264	15,449,290	25,710,039
*Total length ≥ 10,000 bp*	9,664,112	5,805,672	2,525,799
*Total length ≥ 100,000 bp*	0	0	0
*N50*	3,705	6,093	3,278
*N75*	2,514	2,687	2,453
*L50*	3,591	545	2,309
*L75*	7,991	1,611	4,605
*GC%*	62.1	64	58.7
*N’s*	54,379	12,310	27,269
*N’s per 100 kbp*	103.7	79.7	106.1
*Binned contigs*	10,780 (77.6%)	2,870 (86.6%)	6,763 (89.9%)
*Unbinned contigs*	3,118 (22.4%)	446 (13.4%)	762 (10.1%)
*Contigs too short*	0	0	0
*Bins*	11	3	6
*High-quality bins*	0	0	0
*Medium-quality bins*	2	0	0
*Low-quality bins*	11	3	6

**TABLE 3:** Gene counts from whole metagenomes and whole genome sequences that encode for each enzyme.

Enzyme	BPF1 0–30 cm	BPF1 30–58 cm	BPF2 0–30 cm	B3	B4	B5	B7	E5	E6	E7	G16	G17	G19
*α-glucosidase*	0	0	0	0	0	0	0	0	0	0	0	0	4
*β-glucosidase*	8	9	6	0	0	0	0	9	1	0	7	7	9
*Cellubiosidase*	6	6	6	2	1	1	2	2	0	2	2	2	5
*Leucine aminopeptidase*	14	71	4	3	2	2	4	11	7	8	8	8	15
*N-acetyl-β-D-glucosaminidase*	4	4	2	1	1	1	1	2	1	2	1	1	1
*Phosphatase*	24	21	27	10	8	9	12	15	12	13	11	11	15
*Xylosidase*	0	0	6	2	2	2	4	4	1	3	3	3	14

*The list of genes contributing to these counts can be found in Supplementary Table 3.*

## Discussion

### Differentiating Microbial Metabolism Between the Two Active Layer Sites Displayed by Geochemistry

Despite being < 100 m apart, BPF1 and BPF2 differed in the δ^13^C signatures of inorganic carbon and percentages of inorganic carbon, organic carbon, and total nitrogen. The organic carbon δ^13^C of both cores showed signatures originating from plant material (–25 to –35‰) (Mu et al., 2014; Figure 2D). At BPF1, the δ^13^C of inorganic carbon was similar to the δ^13^C of organic carbon at all depths. This likely indicates that the inorganic carbon was produced from organic matter through microbial degradation, since heterotrophy expresses little to no kinetic isotopic effects. In BPF2, the inorganic carbon was ^13^C-enriched ranging from -20 to 14‰ with depth. Three possibilities to explain this are the following: (1) inorganic carbon becomes ^13^C-enriched due to microbial carbon fixation, which can leave behind ^13^C-enriched inorganic carbon due to kinetic fractionation (Boschker and Middelburg, 2002); (2) a large source of ^13^C-enriched inorganic carbon diffuses upward in the core, mixing with ^13^C-depleted heterotrophically produced inorganic carbon as it diffuses downward; and (3) both processes are occurring. Evidence that less microbial heterotrophy may be occurring in BPF2, which would allow autotrophy to have a greater effect on the δ^13^C values for inorganic carbon, comes from the fact that there are lower amounts of labile organic matter, compared to BPF1. C/N ratios can indicate how labile carbon compounds are for microbial activity in the soil, with lower ratios (13–18) indicating a greater availability for metabolic use (Sinsabaugh et al., 2009). BPF1 had significantly more organic carbon (*p* < 0.015) differences by comparable depths between sites and a statistically lower C/N ratio (*p* < 10^–4^), largely driven by the statistically higher nitrogen content (*p* < 10^–5^), suggesting that there may be greater activity of microbial carbon degradation in BPF1 due to higher carbon lability (Figures 2A,G). POXC values, a proxy for organic matter lability (Weil et al., 2003), were also higher in BPF1 (582 mg POXC/g_*dry soil*_ ± 109 vs. 225 mg POXC/g_*dry soil*_ ± 31 for BPF2) (Supplementary Figure 3). Microbial biomass was also greater in BPF1, shown by higher DNA concentrations (Figure 2H, 87% of samples at the same depth were higher in BPF1) and cell counts in the comparable soil increment depths (∼80,000 cells/g soil vs. ∼26,000 cells/g soil, Supplementary Figure 5B and Supplementary Data File 1) than BPF2. However, carbonates could have come from the wide range of carbonate deposits that are found in Svalbard (Koevoets et al., 2016). These could contribute to the proglacial active layer after glacial scouring and would be likely to have values ^13^C-enriched by at least 20‰ relative to organic matter (Kraimer and Monger, 2009), so the possibility of the δ^13^C trends in inorganic carbon could also reflect a carbonate source from below mixing with a heterotrophic source above. Given the evidence for a decrease in heterotrophy in BPF2, and the prevalence of ^13^C-enriched carbonate deposits in the area, both processes likely contribute to the observed stable carbon isotope trends.

Nitrogen is often a limiting nutrient in Arctic environments, and this can reduce the compounds that are available for microbial metabolism (Manzoni et al., 2012; Kou et al., 2020). The inorganic nitrogen at these two sites (NH_4_^+^ and NO_3_^–^) makes up a small portion of the total nitrogen present (<10^–8^%, Figures 2C, 3), which means the limited nitrogen that is present here is organic nitrogen, most likely in the form of plant material and input from animals (Solheim et al., 1996). NH_4_^+^ and NO_3_^–^ are highest in the surface (0.05 and 0.04 μg/g_*dry soil*_, respectively). At both sites and the lowest values of NH_4_^+^ (0.02 μg/g_*dry soil*_) are higher than the highest values of NO_3_^–^ (0.04 μg/g_*dry soil*_; Figure 3). This could be an indication of NH_4_^+^ oxidation or nitrate absorption by plants, since the δ^15^N signature of plant organic matter is 2‰–3‰ (Gauthier et al., 2013). This may occur in the upper few centimeters at both sites, as NH_4_^+^ decreases and NO_3_^–^ slightly increases with depth. Therefore, the δ^15^N signature that is decreasing with depth at both locations likely originates from plant processes and degraded proteins.

Phosphatase generally has the highest activity among the enzymes commonly measured in soils because most soil microbes are capable of creating extracellular phosphatases, and when phosphorus is low, phosphatase enzyme expression is increased (Lee Taylor and Sinsabaugh, 2015). Lastly, the low/below limit of detection values for phosphate in the soil profile (Figure 3) and the increased activities for phosphatase (Figure 5A) further emphasize how these organisms in these soils are limited in nutrients for microbial activity. Even though BPF1 has more labile carbon and higher microbial biomass than BPF2, it only had higher activities in two enzymes (AG and BG) and only in the upper section. Even with less heterotrophy in BPF2, the reason the microbial community maintains similar rates of carbon-degrading enzymes may be to access the nitrogen and phosphorous in organic compounds.

In both Bayelva active layer cores, the highest measured enzymatic activity was for leucine aminopeptidase, and that activity was generally higher at 25°C than at 15 or 5°C. Leucine aminopeptidases are critical cell maintenance enzymes that drive peptide turnover (Matsui et al., 2006) and are often used as an indicator of total peptidase potential in an environment due to the non-specificity of the enzyme (Sinsabaugh et al., 2008). There are many environments that report LAP activity to be the highest among this tested suite of enzymes (Steen et al., 2015). Bacteria have been shown to use leucine, along with other amino acids, as an alternative source of carbon and nitrogen in energy-limited environments (Díaz-Pérez et al., 2016). Nitrogen demand may explain why LAP is nearly two orders of magnitude higher in activity than the other six tested enzymes for both the bulk soil and cultured isolates (Figures 5A,B).

### Soil and Cultured Enzymes Show Cold Adaptation

Characterizing environmental microbes is challenging, as many organisms resist common culturing methods (Lloyd et al., 2018). Our isolates belong to a genus that has previously been found in active layers (Figure 4 and Table 1). *Pseudomonas* species are Gram-negative, aerobic, bacilli organisms (Ramos and Levesque, 2006). This genus contains over 140 species, and most are saprophytic (Ramos and Levesque, 2006) and common in soil. Some *Pseudomonas* sp. can be psychrophilic, while others are mesophilic (Vishnivetskaya et al., 2000; Sonjak et al., 2006; Finster et al., 2009). The isolates we obtained from the Bayelva sites are most closely related to species that have been found from similar cold regions such as Alaska, Antarctica, and Tibetan plateau (Table 1).

A wide range of enzyme classes, including peptidases and carbohydrate lyases, have been shown to be cold adapted (Feller and Gerday, 2003; Kim et al., 2021). One of the best studied enzymes that has a lower K_*m*_ (and therefore greater catalytic efficiency) at cold temperatures is chitobiase, or N-acetyl-β-D-glucosaminidase, obtained from an *Arthobacter* sp. culture (Kim et al., 2021). NAG is also often used as an indicator of nitrogen mineralization within soils (Salmon et al., 2018). This enzyme showed higher V_*max*_ values at 15°C, rather than 25°C in four depths of BPF1 and in five of the *Pseudomonas* sp. cultures. This implies that psychrophily in this class of enzymes may be widespread among active-layer microbes, since multiple isolates and the natural population had this property. The only other enzyme that appeared to be cold adapted was xylosidase, which had its highest V_*max*_ at 15°C in three soil samples and in the same five *Pseudomonas* sp. isolates that had psychrophilic N-acetyl-B-D glucosaminidase. One depth of BPF1 had the highest activity for this enzyme at 5°C. Xylosidase has been found to be cold adapted in a *Duganella* sp. isolate from Antarctic soil (Kim et al., 2021). Both enzymes are good evidence that the cold adaptations observed in pure-culture experiments are upheld in natural microbial populations. This suggests that these enzymes are well adapted to a cold environment but have the flexibility to continue functioning as temperatures warm.

### Metagenomes and Cultures Parallel Enzymatic Results

The read mapping between the metagenomes and the whole genomes of the isolates showed that each isolate was present in the environment (Supplementary Figure 8). However, the low reads per kilobase per million mapped reads (RPKM) values suggests that these organisms were not the dominant community members; instead, they were the best at growing on the general medium used. There were more genes encoding LAP than other enzymes within both the metagenomes and isolates’ whole genomes (Table 3). The second highest gene counts and activity were in PHOS across the whole genomes and the metagenomes. The whole genomes had more gene counts for three of the four carbohydrate-degrading enzymes, (AG, BG, and XYL) than the metagenomes. However, enzymatic activity was often higher in bulk soil than in cultured isolates. This could indicate that microbes in the natural communities have enzymes with higher V_*max*_ values or that the cultured *Pseudomonas* sp. produced lower amounts of enzymes when grown in culture, perhaps because they were not nutrient limited. Additionally, gene presence may not always mean a higher rate of enzymes exported for compound utilization. For instance, leucine can be cleaved by multiple types of enzymes, which may inflate the number of genes for LAP (Steen et al., 2015). This is reflected in the lack of correlation between gene dosage per genome or per metagenome and the measured activity for that enzyme class.

### Ny Ålesund, Svalbard Active Layer Microbes Are Ready for Thaw

Dissolved organic carbon (DOC) is a potential source of carbon and energy for heterotrophic organisms (Kaplan and Newbold, 2000; Fischer et al., 2002). Previous studies have shown high spatial variability in permafrost DOC values. For instance, active layers in the Eight Mile Lake soil in Alaska, United States, ranged from 0.063 to 0.46 mg/g (Waldrop et al., 2010). This location does not reach maximum DOC, 46.38 mg/g, until the bottom of the active layer at 35–45 cm. Comparatively, the DOC in the two Svalbard active-layer sites was highest at the surface (2 mg/g_*soil*_, Figure 3).

Nitrogen is the limiting nutrient for plant growth in arctic tundra, and an increase in nitrogen will increase primary production (Beermann et al., 2017). When the active layer thaws, it provides the rooting zone, which is a region for plant roots and soil microbes to compete for nitrogen (Salmon et al., 2018). Salmon et al. (2018) found that C/N decreased, %N decreased, and %C decreased with depth. The C/N of organic matter is fairly constant with depth at both BPF1 and BPF2, and the range of values from 20 to 28 is lower than the C/N in active layer soil of Eight Mile Lake, Alaska (21–46) (Salmon et al., 2018). This indicates that the increase in nitrogen limitation with depth at the Alaskan site may not apply to our Bayelva, Svalbard active layer samples.

These Svalbard active layer soils differ from Taylor Valley in Antarctic McMurdo Dry Valley soils in DOC, water content, inorganic nitrogen, and labile carbon (Zeglin et al., 2009). Moisture from soils near McMurdo Dry Valley had 20 times the DOC, two times the water content, and three orders of magnitude higher inorganic nitrogen than the Svalbard active layer samples. The McMurdo Dry Valley has a higher inorganic nitrogen contribution from NO_3_^–^, while Svalbard’s inorganic nitrogen contribution is mainly from NH_4_^+^. Additionally, we found the Svalbard active layer to have a higher amount of organic nitrogen. Labile carbon (POXC) in Svalbard was up to a hundred times higher than the Antarctic active layer, even though the DOC was up to 20 times lower. These measurements could indicate that Arctic and Antarctic sites may have different outcomes for carbon degradation as permafrost thaws and microbial activity increases. Our work suggests that Svalbard will have higher activity, compared to McMurdo Dry Valley soils, due to the higher amounts of organic nitrogen and labile carbon based on POXC and C/N ratios.

## Conclusion

The Svalbard active layer soils show unique trends in soil geochemistry between the two sites, despite being only 84 m apart. The two sites show differences in their measured carbon, nitrogen, and lability of the organic carbon present. These differences may be driving the different microbial metabolisms, where BPF1 has an environment and carbon isotopic signatures of microbial heterotrophy, and BPF2 shows evidence that the low activities of heterotrophs allow microbial autotrophy to dominate the isotopic fractionation signal and/or heterotrophically produced inorganic carbon mixes with geological carbonate deposits. BPF1 is on an incline, while BPF2 is in a seasonal river moraine and could be receiving relocated soil material from higher elevations (colluvial soil). These two sites had high values of LAP activity, which is commonly observed in soil environments, perhaps due to the large range of enzymes capable of hydrolyzing leucine (Steen et al., 2015). The four carbohydrate-degrading enzymes, namely, AG, BG, XYL, and CB, had the lowest activity for the soil and the cultures. Carbohydrates often are derived from plants, which are small, scarce, and seasonal in this environment (Figure 1C). Some of these enzymes (XLY and NAG) had higher activity in 5 or 15°C than in 25°C, suggesting that enzymatic adaptation to cold temperatures or an increase in enzyme production may be a strategy for microbial cold adaptation (Zeglin et al., 2009).

The isolates found in the two sites are organisms that have been previously reported in cold soil environments, which suggests that they are from the study site and not contaminants (Table 1). The recruitment of metagenomic reads to the genomes of these isolates further suggests that these 10 *Pseudomonas* sp. are present in the whole *in situ* soil community, but not dominant. While enzymatic activities of bulk soil and isolated cultured are difficult to compare to true *in situ* environmental activities, there was agreement between the two. The culture and bulk soil enzymatic activities had highest values in LAP. PHOS activities were all higher in the 25°C, while XYL and NAG showed some higher activities in the lower temperatures tested. The total cell counts for the bulk soil were an order of magnitude larger than the cultures. The cultures cell counts ranged from 100 to ∼36,000 cell/ml, and the bulk soil ranged from 10,000 to 180,000 cells/g_*soil*_ (Supplementary Figures 5A,B and Supplementary Data File 1). Despite this, their potential maximum enzymatic activities were similar. This could mean that a smaller number of organisms are able to have the same enzymatic effect as a larger population.

Given the greater activity of most enzymes at higher temperatures, it is likely that Svalbard active-layer soils will experience higher microbial activity as the temperatures increase in this warm permafrost. From the data presented here, it is likely that the microbes will become more active once the Svalbard active layer expands as compared to the microbes present in Taylor Valley of McMurdo Dry Valley sites (Zeglin et al., 2009). The measured higher quantity of labile carbon in areas of soil like BPF1 could indicate that microbial activity will spike during permafrost thaw and active layer expansion. While nitrogen is still a limiting nutrient for microbial activity in the Arctic, the increase in soil organic matter degradation will introduce more labile nitrogen and carbon compounds, allowing for a higher rate of microbial activity. If microbial respiration increases, then stored nitrogen will be more available for plants to grow and perhaps could further lead to higher rates of plant derived carbon enzyme activity. This study demonstrates that the active layer soil near Bayelva in Ny Ålesund, Svalbard will become more microbially active with different carbon degradation pathways, adaptable enzymatic activities, and utilization of the scarce resources.

## Data Availability Statement

Whole genomes and Genbank submissions can be found on NCBI accession number PRJNA649544 and MZ773212–MZ77322, respectively. Kbase with the workflow of the metagenomic analysis can be found on permanent links: WGS: https://narrative.kbase.us/narrative/83182 MISEQ; https://narrative.kbase.us/narrative/56628.

## Author Contributions

KS and RP contributed to the idea and hypothesis generation, experimental design, computational work, writing, and editing the manuscript. AF and PL contributed with experimental assistance and editing the manuscript. RL, JB, and SS edited the manuscript. TO, TV, and KL were responsible for funding acquisition and editing the manuscript. KL contributed by advising the direction of the project. All authors contributed to manuscript revision, read, and approved the submitted version.

## Conflict of Interest

The authors declare that the research was conducted in the absence of any commercial or financial relationships that could be construed as a potential conflict of interest.

## Publisher’s Note

All claims expressed in this article are solely those of the authors and do not necessarily represent those of their affiliated organizations, or those of the publisher, the editors and the reviewers. Any product that may be evaluated in this article, or claim that may be made by its manufacturer, is not guaranteed or endorsed by the publisher.
