# Supplementary material for: Permafrost Active Layer Microbes From Ny Ålesund, Svalbard (79°N) Show Autotrophic and Heterotrophic Metabolisms With Diverse Carbon-Degrading Enzymes
Source: Front Microbiol. 2022 Feb 3;12:757812. doi: 10.3389/fmicb.2021.757812 (PMC8851200; doi:10.3389/fmicb.2021.757812)
Supplement: Supplementary file 2 [file Data_Sheet_1.PDF]

For Svalbard Active Layer Paper, Sipes and Paul et al.

Supplemental Figures and Tables

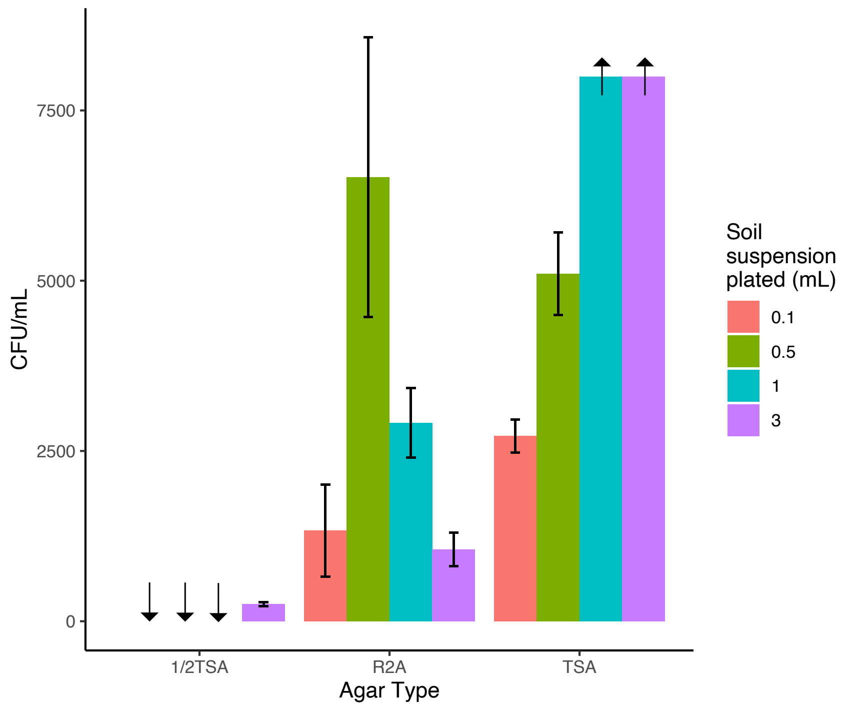

Figure S1: Preliminary testing for mean CFU/mL from agars ½ TSA, R2A, and TSA with surface soil from BPF2. Downward-facing arrows indicate samples that were too low to count, upward-facing arrows indicate samples that were too high to count.

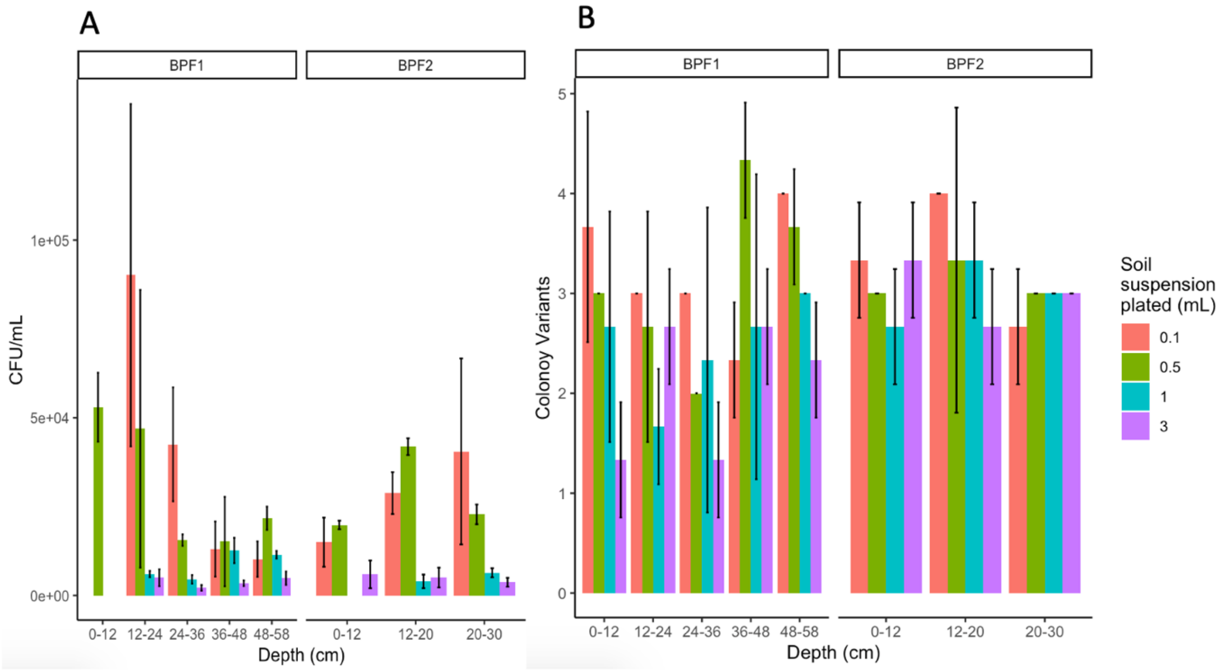

Figure S2: Each interval from BPF1 and BPF2 were plated on R2A agar using the multiple dilution schemes. R2A plates were incubated at 4°C for at least three weeks. **A)** Mean CFU/mL values on each of the depth segments. **B)** Unique colony variations between the depth segments.

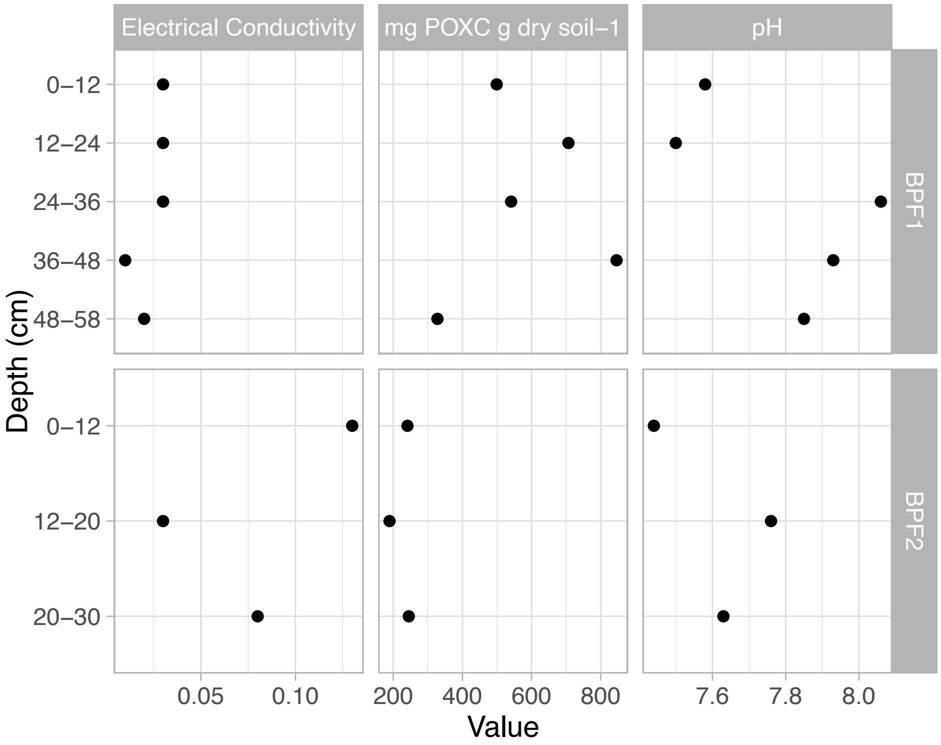

Figure S3: Electrical conductivity, permanganate oxidizable carbon (POXC) for labile carbon, and pH for each soil interval.

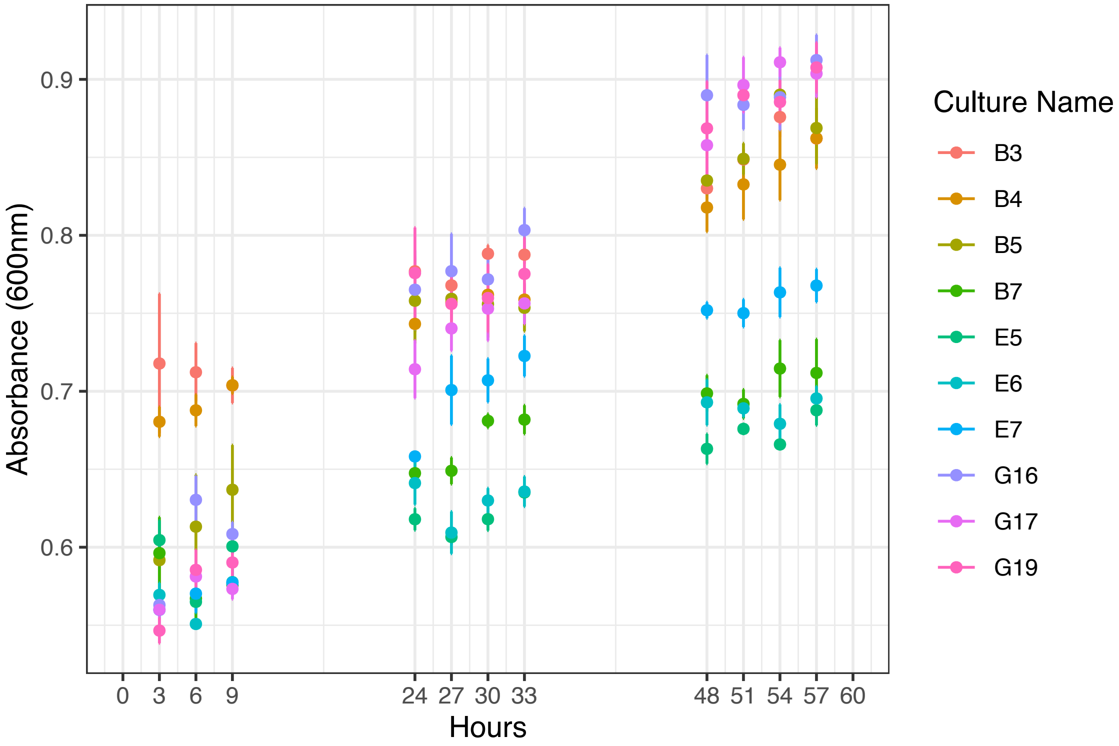

Figure S4: 60-hour growth curve for the 10 isolates in R2A broth at 25°C.

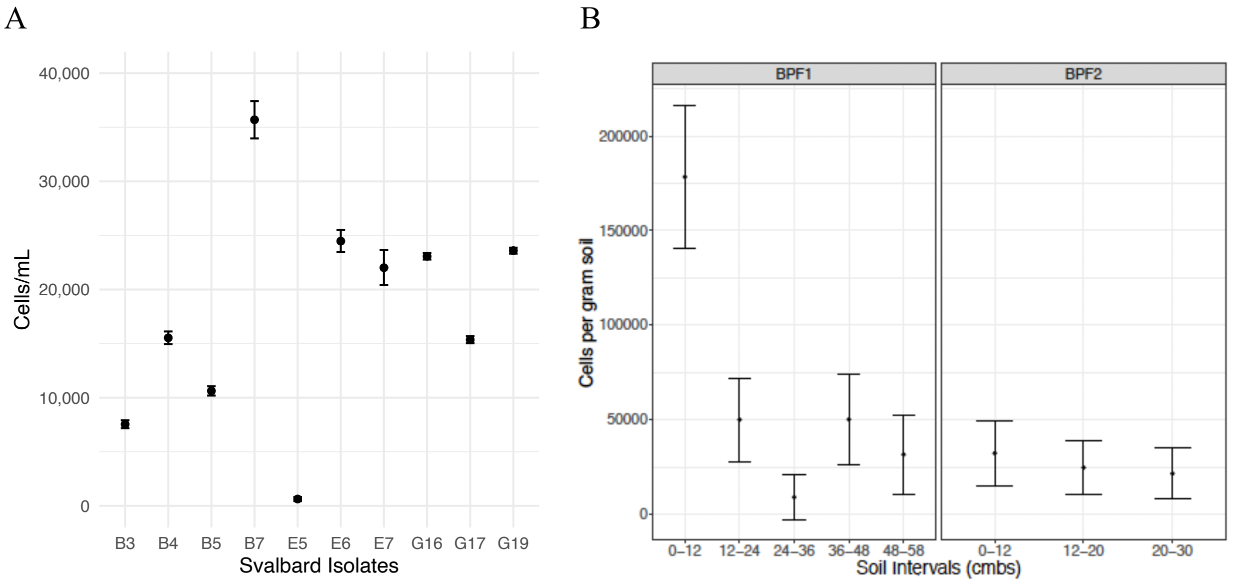

Figure S5: **Direct microscopic cell counts** in A) 1mL of culture broth of Svalbard isolates, and B) Bulk soil. Cells were stained with SYBR Gold and 30 random fields of view were averaged for each sample. Error bars show one standard deviation about the mean.

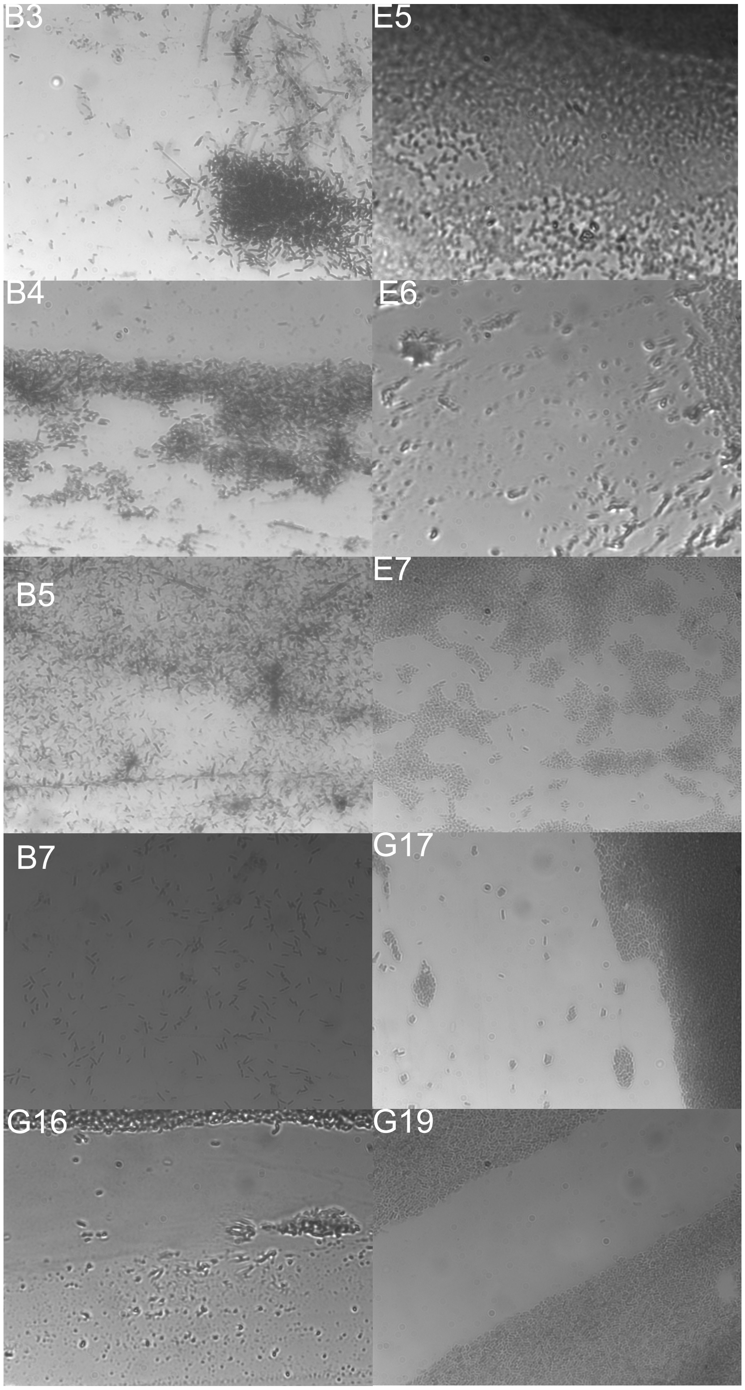

Figure S6: Each isolate at 10X under a Zeiss X2 Imager Microscope.

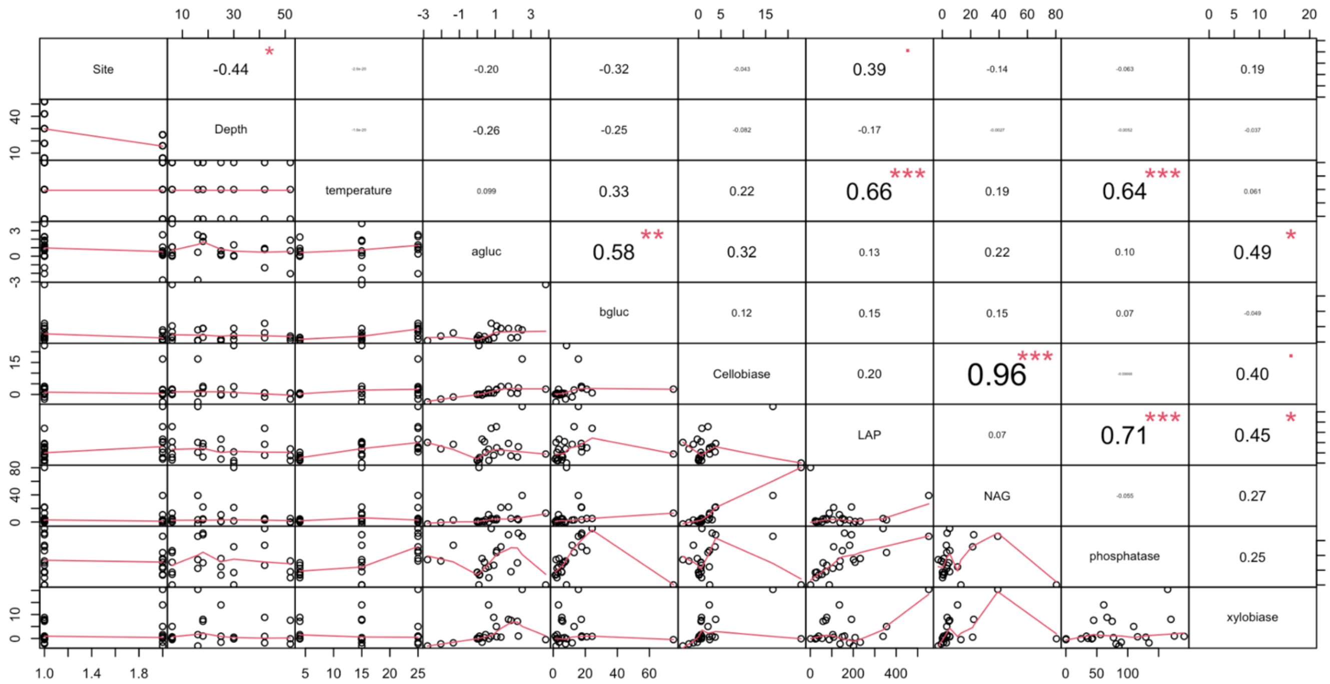

Figure S7: Spearman correlations between all variables in the enzymatic activity experiment: Site, depth, temperature and the seven enzymes. The left side of the diagonal of the graph is the distribution of the corresponding variable as the x (above the graph) and the y (right of the graph). The correlation coefficient is reported by the number in the boxes in the right side of the diagonal. The red stars indicate p values of *=0.05, **=0.01, ***=0.001.

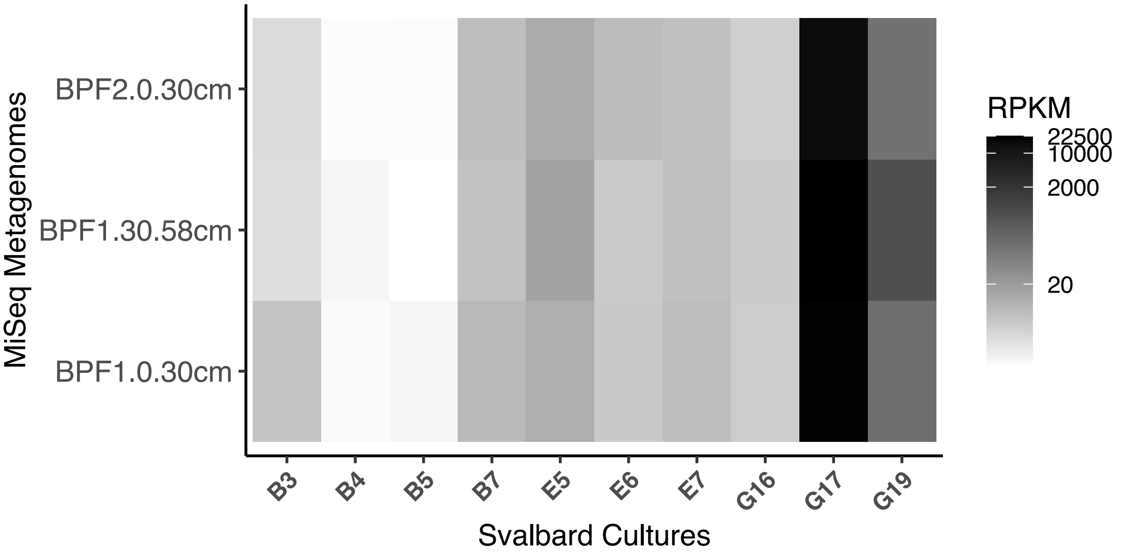

Figure S8: Read mapping done in reads per kilobase per million mapped reads (RPKM) between the Whole Genome Sequenced Isolates and the MiSeq Metagenome Libraries.

A

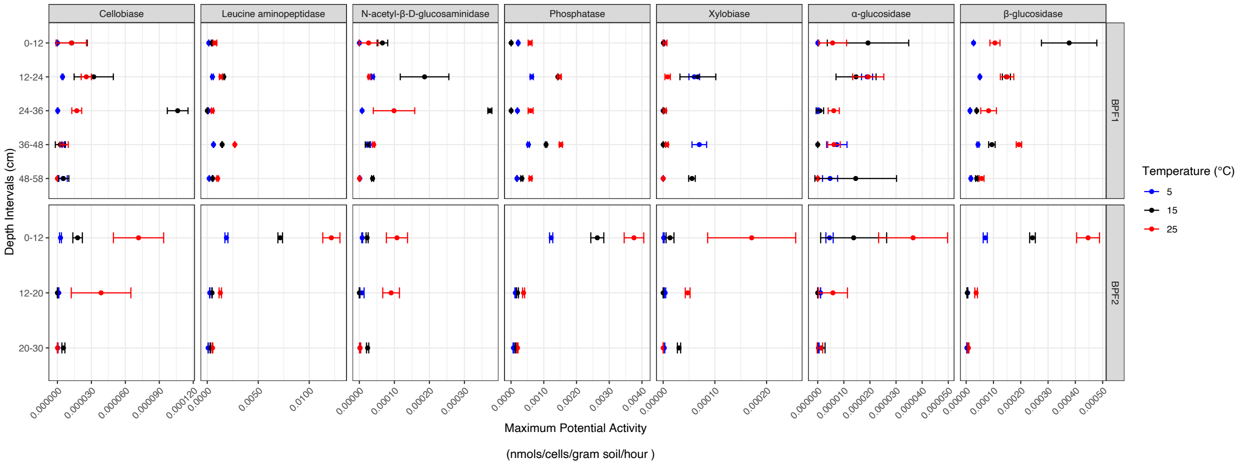

B

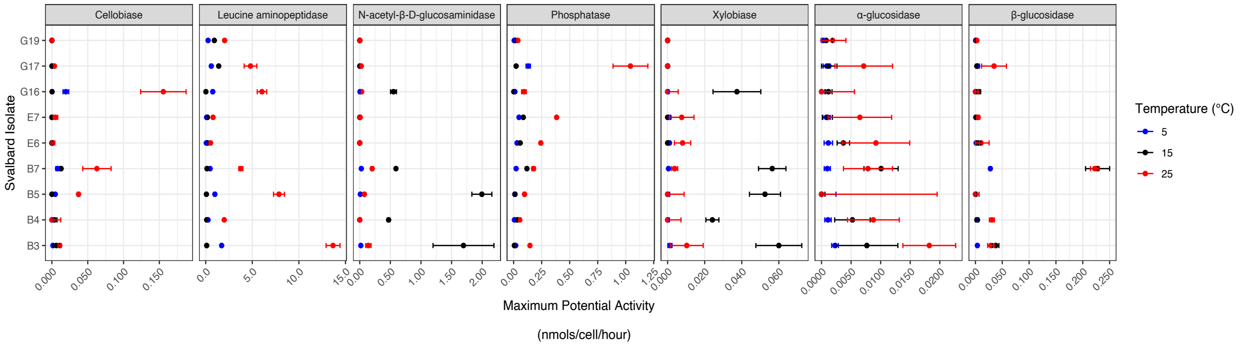

Figure S9: Enzyme activity was done in triplicate measurements at each temperature (color differences) for all samples. **A)** Core site interval depths. **B)** 10 cultured isolates. Each enzyme's potential activity is plotted within its own detected range at nmols/sample/hour and normalized base on cell counts (see cell count supplemental methods) for each method. Negative activity values were determined to be zero.

Table S1: Bulk soil average enzyme activity in nmol g dry soil^{-1} hr^{-1}. AG; α-glucosidase, BG; β-glucosidase, CB; Cellubiosidase, LAP; Leucine aminopeptidase, NAG; N-acetyl-B-D-glucosaminadase, PHOS; Phosphatase, XYL; Xylosidase

Sample	°C	CB	LAP	XYL	AG	BG	NAG	PHOS
BPF1 0-12	4	-0.01891634	14.62690496	0.06712252	-0.000610205	2.610760061	0.05	43.13
BPF1 12-24	4	0.001571204	0.174834495	0.02098741	0.006594753	0.017448974	4.54	74.93
BPF1 24-36	4	3.93565E-05	0.332830972	-0.002263	-3.57181E-17	0.042898627	1.67	41.96
BPF1 36-48	4	0.002842535	0.336585927	0.03858498	0.004044226	0.023887859	3.42	66.59
BPF1 48-58	4	0.175759748	6.618544147	-0.0205911	0.167670387	0.603024985	0.16	24.50
BPF1 0-12	15	2.314098312	82.56388243	-0.4239038	3.571911254	69.95803283	13.08	0.08
BPF1 12-24	15	0.001014161	0.051088281	0.00212189	0.000464075	0.004664384	22.12	169.85
BPF1 24-36	15	0.940767113	0.042647753	-0.00404693	-3.57181E-17	0.340664943	80.45	0.30
BPF1 36-48	15	-0.0031029	0.481967099	-0.00475387	-0.003528958	0.031168132	2.83	134.37
BPF1 48-58	15	0.002586361	0.310925698	0.03289366	0.00815832	0.022739152	5.14	44.90
BPF1 0-12	25	0.004005994	0.245156642	0.00118744	0.001787691	0.034435884	5.25	115.94
BPF1 12-24	25	0.005146887	0.270595539	0.00174025	0.003900555	0.03021199	3.36	175.41
BPF1 24-36	25	0.00133276	0.039399493	0.00020545	0.000479372	0.00642148	21.38	129.23
BPF1 36-48	25	0.000752293	0.345531298	0.00090902	0.000797073	0.024754033	5.13	191.20
BPF1 48-58	25	0.034950642	-2.243781606	0.03614213	0.032964809	-0.124710218	-0.76	81.22
BPF2 0-12	4	0.009333702	6.486834494	0.00339629	0.01563761	0.241405681	0.24	35.78
BPF2 12-20	4	0.002694385	0.568340866	0.00875777	0.002515573	0.012870465	3.28	56.14
BPF2 20-30	4	0.004703593	1.632476249	0.04018685	0.004868634	0.052399687	0.72	33.20
BPF2 0-12	15	0.010434534	4.189273462	0.00777444	0.008059451	0.14229918	0.65	76.93
BPF2 12-20	15	0.014199689	-0.809565853	0.01241964	0.011199037	-0.007110017	-2.90	85.79
BPF2 20-30	15	0.003631818	0.205756807	0.02122036	0.000956156	0.005028672	10.75	61.27
BPF2 0-12	25	0.006086564	1.033641051	0.01454205	0.003103802	0.037937288	3.13	109.63
BPF2 12-20	25	0.002584444	0.085782999	0.0031755	0.000390073	0.002441777	39.10	164.57
BPF2 20-30	25	-0.00346513	2.713889143	-0.01810231	0.003106666	0.042537433	0.96	89.51

Table S2: Culture average enzyme activity in nmol ml^{-1} hr^{-1}. AG; α-glucosidase, BG; β-glucosidase, CB; Cellubiosidase, LAP; Leucine aminopeptidase, NAG; N-acetyl-B-D-glucosaminadase, PHOS; Phosphatase, XYL; Xylosidase

Sample	°C	AG	BG	CB	LAP	NAG	PHOS	XYL
B3	5	5.889E-05	9.343E-05	3.538E-05	4.416E-02	4.552E-04	4.701E-04	3.342E-05
B4	5	5.837E-05	2.041E-04	-3.852E-04	1.322E-02	-1.535E-04	3.647E-04	-2.478E-04
B5	5	-9.722E-05	-2.794E-05	1.692E-04	3.581E-02	3.440E-04	4.429E-04	-7.986E-05
B7	5	1.201E-04	3.401E-03	9.608E-04	5.764E-02	3.106E-03	2.713E-03	5.822E-05
E5	5	0.0001213	6.7017E-05	3.5969E-05	0.00650309	0.00013235	0.00388765	2.073E-05
E6	5	9.649E-05	1.119E-04	1.265E-05	5.423E-03	3.963E-06	2.539E-03	8.462E-05
E7	5	6.120E-05	4.798E-05	4.670E-05	7.436E-03	6.354E-05	3.617E-03	7.175E-05
G16	5	-1.399E-04	-1.315E-04	1.538E-03	5.970E-02	3.001E-04	9.094E-04	-1.442E-04
G17	5	4.695E-05	2.281E-04	2.328E-05	3.164E-02	1.610E-04	6.859E-03	-1.876E-05
G19	5	1.905E-05	2.821E-05	-1.738E-05	2.054E-02	4.570E-05	3.840E-04	-3.008E-05
B3	15	4.9558E-05	0.00024412	3.9481E-05	0.00068525	0.01095167	4.2243E-05	0.00038771
B4	15	6.9572E-05	3.5669E-05	4.8562E-05	0.00139917	0.00627899	0.00043975	0.00032164
B5	15	-9.328E-05	3.8003E-06	-5.737E-05	0.00068897	0.01816254	0.00010051	0.00047831
B7	15	0.0003081	0.00696319	0.00039172	0.0041447	0.01807888	0.00362262	0.0017266
E5	15	2.9345E-05	0.00012179	6.9347E-06	0.00720835	-3.654E-05	0.00115197	0.00010263
E6	15	7.7473E-05	0.00012476	-3.65E-05	0.00432777	-5.304E-05	0.00122157	-8.357E-05
E7	15	1.9308E-05	2.6815E-05	-1.094E-05	0.00348942	2.6143E-05	0.00162423	-1.408E-05
G16	15	2.3261E-05	0.0001138	3.5238E-06	0.00040202	0.01090368	2.5603E-05	0.00073921
G17	15	1.641E-05	3.3509E-05	-9.295E-06	0.01834945	1.8086E-05	0.00029233	-9.137E-05
G19	15	1.6029E-05	2.3596E-05	-1.312E-05	0.01871441	1.4081E-05	0.00033731	-3.529E-05
B3	25	1.7005E-05	2.1256E-05	5.2323E-05	0.00618252	2.9759E-05	0.0003963	-2.191E-05
B4	25	0.00011102	0.00024589	-0.0001608	0.00959177	-2.009E-05	0.00077357	0.00011966
B5	25	5.3883E-05	8.9426E-05	-0.0001808	0.00493833	8.84E-05	0.00048847	7.2908E-05
B7	25	4.2544E-05	0.00184824	0.00117401	0.01776767	0.00154004	0.000628	0.0001662
E5	25	3.9015E-05	3.0836E-05	3.0413E-05	0.00881604	6.6514E-05	0.00145725	1.2786E-05
E6	25	-1.246E-05	3.3657E-05	-1.217E-05	0.00477814	4.1836E-05	0.00124977	-2.416E-05
E7	25	2.3731E-05	5.5964E-05	1.5751E-05	0.01315247	7.8339E-05	0.00138254	0.00014377
G16	25	7.9532E-05	8.1324E-05	2.6517E-05	0.01605988	4.2929E-05	0.00076292	4.338E-05
G17	25	4.7426E-05	9.8148E-05	0.00012399	0.00632102	0.00011569	0.00068852	0.00044066
G19	25	4.6859E-05	5.8478E-05	7.6177E-05	0.00772018	3.8212E-05	0.00072055	1.0921E-05

Table S3: List of the gene names from UniProt and or CAZY that were used in the total counts for the metagenome and whole genome isolates.

Gene product name	Enzyme
cellobiose phosphorylase	Cellubiosidase
Cellobiose 2-epimerase	Cellubiosidase
pepA/Cytosol aminopeptidase	Leucine aminopeptidase
Putative Cytosol aminopeptidase	Leucine aminopeptidase
Aminopeptidase	Leucine aminopeptidase
Putative lipoprotein aminopeptidase LpqL	Leucine aminopeptidase
Cytosol Aminopeptidase	Leucine aminopeptidase

Aminopeptidase N	Leucine aminopeptidase
Dipeptidyl aminopeptidase 4	Leucine aminopeptidase
D-aminopeptidase	Leucine aminopeptidase
Dipeptidyl aminopeptidase BIII	Leucine aminopeptidase
Dipeptidyl aminopeptidase BII	Leucine aminopeptidase
Dipeptidyl aminopeptidase BI	Leucine aminopeptidase
N-acetylglucosaminyldiphosphoundecaprenol N-acetyl-beta-D-mannosaminyltransferase	N-acetyl-B-D-glucosamididase
tagA	N-acetyl-B-D-glucosamididase
N-acetyltransferase domain-containing protein	N-acetyl-B-D-glucosamididase
Glycoside hydrolase superfamily	N-acetyl-B-D-glucosamididase
N-acetyl-beta-D glucosaminidase	N-acetyl-B-D-glucosamididase
Beta-N-acetylglucosaminidase	N-acetyl-B-D-glucosamididase
Nag	N-acetyl-B-D-glucosamididase
Phosphoserine phosphatase 1	Phosphatase
Phosphoglycolate phosphatase	Phosphatase
Alkaline phosphatase D	Phosphatase
Alkaline phosphatase	Phosphatase
Phosphoadenosine phosphosulfate reductase	Phosphatase
Phosphoserine phosphatase RsbU	Phosphatase
Phosphoserine phosphatase SerB2	Phosphatase
Endo-1,4-beta-xylanase A/xynA1	Xylosidase
Beta-xylosidase/xynB	Xylosidase
Bifunctional xylanase/deacetylase/xynD	Xylosidase
Xyloglucanase	Xylosidase
Beta-xylosidase	Xylosidase
Glucan 1,4-alpha-glucosidase SusB	Alpha glucosidase
Beta-glucosidase BoGH3A	Beta glucosidase
Thermostable beta-glucosidase B	Beta glucosidase
Beta-glucosidase A	Beta glucosidase
Beta-N-acetylglucosaminidase/beta-glucosidase	Beta glucosidase
Beta-glucosidase B	Beta glucosidase
Glucan endo-1,3-beta-glucosidase A1	Beta glucosidase
Beta-glucosidase BoGH3B	Beta glucosidase
Beta-glucosidase	Beta glucosidase
putative 6-phospho-beta-glucosidase	Beta glucosidase
Oligo-1,6-glucosidase	Beta glucosidase
cellobiose phosphorylase	Cellubiosidase
Cellobiose 2-epimerase	Cellubiosidase
pepA/Cytosol aminopeptidase	Leucine aminopeptidase
Putative Cytosol aminopeptidase	Leucine aminopeptidase
